# Science’s temporary workforce: The use of temporary contracts in research has reached a crisis point in Italy but the problem is prevalent across Europe

**DOI:** 10.1038/s44319-026-00847-9

**Published:** 2026-06-24

**Authors:** Marta Paterlini

**Affiliations:** Karolinska Institutet, Stockholm, Sweden

**Keywords:** Careers, Economics, Law & Politics, Science Policy & Publishing

## Abstract

Italy’s post-pandemic recovery plan created thousands of new research positions and raised hopes of more sustainable careers. As the first contracts expire, however, longstanding questions about career structures and research funding have re-emerged in Italy and across Europe.

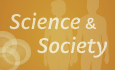

When Italy’s recovery funds arrived during the COVID-19 crisis, they were supposed to do more than just keeping laboratories afloat. In 2021, the National Recovery and Resilience Plan (PNRR), funded through the EU’s Next Generation programme, the recovery initiative set out by the European Commission in response to the COVID-19 pandemic, was supposed to be a turning point: a chance to transform temporary hiring of scientists into more durable positions. The plan, overseen by the Ministry of University and Research, channelled more than €11 billion into research, alongside broader investments in education, innovation and research infrastructure, but scientists were already asking back then whether it would lead to structural change. A few years later, many of the researchers hired under that plan say they are facing the same cliff edge as before, only higher.

## A fragile basis of short-term contracts

Italy’s research system had long been built on a fragile base of temporary appointments, fragmented funding and limited career paths. The PNRR did not create that structure, but injected an unprecedented influx of funding into it, rapidly expanding hiring but without fundamentally changing academic career paths. At the Consiglio Nazionale delle Ricerche (CNR), Italy’s largest public research organisation, Mariacristina Gagliardi describes what happened when the money and the promise of long-term career prospects ran out. She joined CNR in Pisa in 2019 on an *assegno di ricerca* (research fellowship), a weakly protected contract that was later abolished. “When the PNRR funding arrived, it brought a new wave of hiring: positions that began in October 2023 and ended on 30 April 2026,” she said, leaving many young researchers with uncertain career prospects. “The uncertainty is not only about funding but about identity: you are hired as part of a system that does not clearly define what you are supposed to become,” Gagliardi added. She and many other CNR researchers call it “a great hope dissolved”. The ongoing stabilisation process is far too limited: according to unions and researchers involved in the process, it covers around 185 positions nationwide for about 854 eligible candidates. Entire lines of research—from diagnostics to food-chain science—risk disappearing as projects lose staff as quickly as they had gained them.

Italy’s research system had long been built on a fragile base of temporary appointments, fragmented funding and limited career paths.

Organisations representing early-career researchers, such as ADI (Associazione Dottorandi e Dottori di Ricerca in Italia), have long criticised that the Italian system relies on temporary contracts as a substitute for stable employment. Davide Clementi, ADI national secretary, said the core problem is that Italy has never moved beyond the employment model created by the 2010 university reform, which normalised temporary work and made it a structural feature of the Italian research system. The research fellowship in particular became a tool that was not just used but abused. The contract offered few benefits: no unemployment coverage, no sick leave, no holidays. In ADI’s 2025 postdoc survey, 67% of 2888 respondents were employed on research fellowships with limited benefits, and 33.7% said their position would expire between August 2025 and July 2026. ADI argues that recent government measures bring only limited improvements—modest pay increases and inflation indexation—for a small group, but leave the underlying problem unresolved.

The discontent has become visible in the streets as well. In December 2025, precariously employed researchers occupied the CNR headquarters in Rome, pitching tents and holding a permanent assembly. Antonio Musarò, a professor at Sapienza University in Rome, argues that Italy has tried to fix precarity by changing contract types rather than addressing the underlying problem of limited contracts. The new research contracts introduced after the abolition of the research fellowship provide more protections—parental leave, sick pay, pension contributions—but they are also significantly more expensive. What once cost roughly €24,000 a year for a research fellow can now reach €80,000 over 2 years for a contract researcher. The result is better contracts, but fewer of them. “We thought we were solving precarity by eliminating precarity,” Musarò commented.

In December 2025, precariously employed researchers occupied the CNR headquarters in Rome, pitching tents and holding a permanent assembly.

## Attempts to stabilise career paths

In June 2025, the Chamber of Deputies approved two new instruments—“research assignments” and “post-doc assignments”—to create a more reliable early-career pathway, but these are only partial remedies. At the same time, Italy continues to train far more researchers than it retains. During the past decade, each year around 14,000 scientists have left the country after training along with about 150,000 graduates overall. In 2023, Italy had about 173,000 researchers—significantly fewer than France (around 340,000) or Germany (over 459,000), despite recent efforts to expand recruitment. The PNRR represented a rare moment of expansion, but without structural reform, this momentum is now deflating again (Ciriminna and Pagliaro, 2023). Luca Bonini, a neuroscientist at the University of Parma, says that many of the PNRR-funded hires were temporary junior posts created by short-term funding, not by a long-term plan. In retrospective, he believes it was propably a mistake to recruit such a large number simply because the resources temporarily made it possible. “Certainly, much of the problem originated upstream,” he stated. “At this point, I do not think there are many real solutions to the situation that has been created.”

In March 2026, the Ministry of University and Research (MUR) signed a decree on its *Extraordinary Plan for the Recruitment and Enhancement of Research Personnel*, allocating €18.5 million in 2026 and €60.7 million annually from 2027 on to support the hiring of up to 2000 researchers, 1051 of them linked to PNRR projects. The ministry presents the measure as part of a broader strategy that includes a new 3-year research plan, a €300 million continuity fund and a fixed timetable for funding calls. The continuity fund, introduced through the 2025 Budget Law, will be distributed through performance-based assessments of the National Centres and Extended Partnerships. “The measures introduced by the ministry outline a clear strategy: more resources, more stable rules, longer-term planning and greater opportunities for researchers,” a ministry spokesperson said. “The aim is to make the Italian research system stronger, more attractive and more closely aligned with international standards.”

But the criticism does not abate: that this is more of a “bridge” measure, rather than a genuine structural reform. Around 4500 fixed-term researchers will see their contracts expire between 2025 and 2026; even in the best case, fewer than half will find a stable position. At the CNR, as of December 2024, 2939 workers were considered eligible for stabilisation under existing procedures, while the so-called Lotito amendment brings enough funding for another 48 researcher positions—a small fraction of the institute’s fixed-term PNRR workforce. Moreover, co-financing requirements, whereby universities cover up to 50% of the cost risk, benefit only the strongest institutions, leaving behind those in more fragile financial conditions, where precarious research employment is common.

## Different versions of the same problem

The Italian case is not unique though. Across Europe, different systems produce different versions of the same structural problem. Sebastian Kubon at the University of Hamburg and one of the voices behind the #IchBinHanna campaign—a grassroots movement protesting the widespread use of temporary contracts in academia—identified to the *Wissenschaftszeitvertragsgesetz* as the underlying problem. This 2007 law allows fixed-term contracts for up to 6 years before the doctorate and for another 6 years after. Most of these contracts are attached to grants, so when the funding ends, the job ends.

Kubon argues that the law itself normalises precarity and is ultimately damaging for research. “That view is also backed by research” he commented, “A European study found that academics on permanent contracts report higher job satisfaction than those on temporary contracts, and an OECD review notes that academic careers increasingly rely on precarious contracts while rewarding research output over teaching and other duties.”

Ulrich Dirnagl, former Director of the Department of Experimental Neurology at Charité–Universitätsmedizin Berlin, who has long criticised Germany’s academic career structure, argues that the problem extends beyond the law itself. “The Mittelbau is very small,” he said, referring to the permanently employed mid-level academic staff that provide continuity within departments. Most research positions, he notes, are externally funded and therefore tied to projects, a trend reinforced over the years by large competitive funding programmes. The result, he explained, is that researchers move from contract to contract during the very years in which they are trying to build stable lives and families. In his view, this is not something individual universities can solve on their own, but would require system-wide reform—something he says there is little political appetite for.

… researchers move from contract to contract during the very years in which they are trying to build stable lives and families.

Andrea Musacchio, Director at the Max Planck Institute for Molecular Physiology, added a nuance: permanent posts are necessary for permanent tasks, yet a system with no turnover can become closed and unable to renew itself. At his institute, only a limited number of staff scientist roles can be permanent. “If you make everything permanent,” he commented, “you lose the ability to renew.”

… permanent posts are necessary for permanent tasks, yet a system with no turnover can become closed and unable to renew itself.

Other countries have taken different routes to solve the problem of precarious career paths based on time-limited contracts. In Sweden, some fixed-term contracts can convert to permanent ones after 12 months under the Employment Protection Act. Swedish unions and sector voices have long argued for more permanent posts and clearer career paths, while others warn that too much rigidity can make staffing harder in small or unevenly funded institutions. Nonetheless, the Swedish Higher Education Authority reported that around 32% of university staff were employed on fixed-term contracts in 2022—roughly double the rate in the wider labour market.

Switzerland is trying to address the problem at the level of funding itself. In 2025, the Swiss National Science Foundation (SNSF) announced a reform of its career funding schemes: grantees will be employed directly by a Swiss host institution, giving them access to social security and other benefits that traditional fellowships often lack. The SNSF also said eligibility criteria would rely less on rigid timelines and more on career stage, acknowledging that academic careers have become increasingly non-linear. The reform does not eliminate temporary employment, but it reflects a broader recognition across Europe that the structure of research careers—not only the level of funding—contributes to job insecurity.

France follows a different model: instead of a long and protracted postdoc bottleneck, it offers more permanent positions through competitive examinations. The system offers stability for those who secure a permanent post, but it is highly selective at the point of entry. According to the French Ministry of Higher Education and Research, 92% of scientists from the 2018 cohort were employed 3 years after their doctorate, 67% of those had stable jobs, and 62% were still working in research.

In the UK, short-term contracts are not tied to a specific law, but to the structure of research funding itself. As Sara Shinton, Director of the UKRI (UK Research and Innovation) *Future Leaders Fellows Development Network*, explains, precarity is “a fundamental feature” of the academic career, because funding is project-based for limited periods. There are longer-term models—particularly in research institutes—but even these typically extend to just a decade rather than an entire career. Efforts have been made to mitigate the effects. National frameworks such as the Concordat for Career Management of Research Staff, along with initiatives like the Prosper project at the University of Liverpool, aim to help researchers plan beyond the postdoc. One alternative model would be for universities to retain pools of experienced researchers who can move between projects, providing continuity without tying individuals to a single grant. But such models require institutions to share financial risk—something they have been reluctant to do. “There is no risk appetite,” Shinton said.

## A EU-wide solution?

At the European level, the problem has not gone unnoticed. According to a Commission official involved in the European Research Area (ERA), researchers’ precarity has emerged repeatedly in stakeholder consultations for the upcoming ERA Act. “The Commission is currently exploring what concrete measures can fit into this legislative context, which aims at contributing to the implementation of a fifth freedom for researchers and knowledge, in line with the Letta report,” the official said.

One existing instrument already sets a bar. The Marie Skłodowska-Curie Actions (MSCA) programme requires that its beneficiaries—whether doctoral networks, postdoctoral fellowships or COFUND projects—work under an employment contract with full social-security benefits: sickness and parental, leave unemployment and invalidity benefits, pension rights and accident protection. That includes doctoral candidates, who in many countries are still treated as trainees rather than workers. The programme also offers competitive living allowances adjusted to national costs, plus mobility, family and special needs allowances. Yet the MSCA covers only a fraction of Europe’s research workforce, and the proposed ERA Act—still under discussion—would need to translate these principles into binding rules across national systems. Whether it will succeed where voluntary charters have largely failed is an open question.

Across these different national contexts, a common pattern emerges. Italy’s problem is the cliff edge created by a temporary funding boost. Germany has a legal framework that enforces early exit. The UK’s funding model normalises short-term employment. But in all cases, the underlying tension is the same: research depends on long-term thinking, while the systems that fund it operate on short-term cycles. That tension shapes not only careers, but the direction of science itself—pushing researchers toward publication-driven incentives and short-term metrics rather than slower, more careful research cultures, as Dirnagl argued.

But in all cases, the underlying tension is the same: research depends on long-term thinking, while the systems that fund it operate on short-term cycles.

The PNRR was, in principle, an opportunity to change that: to turn temporary expansion into structural reform. Instead, it has highlighted the limits of a model that creates positions without creating careers. As the first wave of contracts approaches its end, Italy is once again facing the question it has long postponed: what happens after the project ends? Several years after the launch of the recovery plan, that question remains largely unanswered.

## Supplementary information

Peer Review File
