# Supplementary material for: Science’s temporary workforce: The use of temporary contracts in research has reached a crisis point in Italy but the problem is prevalent across Europe
Source: EMBO Rep. 2026 Jun 24;27(14):3909–11. doi: 10.1038/s44319-026-00847-9 (PMC13400635; doi:10.1038/s44319-026-00847-9)
Supplement: Supplementary file 1 — Peer Review File [file 44319_2026_847_MOESM1_ESM.pdf]

EMBO reports

# Science's temporary workforce

Marta Paterlini

*Corresponding author(s): Marta Paterlini (martapaterlini@gmail.com)*

## Review Timeline:

| | |
|---|---|
| Submission Date: | 12th Jun 26 |
| Accepted: | 15th Jun 26 |

*Editor: Holger Breithaupt*

## Transaction Report: This article was editorially reviewed.
